# The Role of Dietary Schizochytrium Powder in Chicken Production Performance, Egg Quality, and Antioxidant Status

**DOI:** 10.3390/ani15233494

**Published:** 2025-12-04

**Authors:** Qianbao Wang, Huayun Huang, Chunmiao Li, Zhengyang Huang, Zhaolin Wu, Linglin Kong, Zhenhua Zhao, Zhicheng Wang

**Affiliations:** Jiangsu Institute of Poultry Science, Yangzhou 225125, China; wqb15855142436@163.com (Q.W.); huanghuayun520@163.com (H.H.); cherrylcm@163.com (C.L.); hgyjips@126.com (Z.H.); wzl13705258141@163.com (Z.W.); klljips@126.com (L.K.)

**Keywords:** animal nutrition, poultry, dietary supplementation, performance, antioxidant function

## Abstract

Objectives: The aim of this experiment was to explore how the addition of Schizochytrium powder to the feeding ration affected the production performance, egg quality, and antioxidant function of chickens. Schizochytrium powder is a unicellular spherical marine microalga that can be cultivated through heterotrophic fermentation, with characteristics including rapid cell growth, stable composition, and ease of large-scale production. Experimental design: Three hundred and twenty 33-week-old chickens with similar egg production rates and body weights were selected and randomly divided into four groups, with five replicates each and 16 hens in each replicate. The control group (Group I) was fed a corn–soybean meal basal diet, while the test groups were supplemented with 0.5% (Group II), 1.0% (Group III), and 2.0% (Group IV) Schizochytrium powder on top of this basal diet, respectively. The pretest period was 1 week, and the main test period was 8 weeks. The results revealed the following: (1) Compared with Group I, the average daily feed intake (ADFI) and laying rate (LR) were significantly lower (*p* < 0.05) in Group IV, and there was no significant difference (*p* > 0.05) in the ADFI and LR between Groups II and III. (2) Compared with Group I, the eggshell strength (ES) and docosahexaenoic acid (DHA) content of Groups II, III, and IV were significantly higher (*p* < 0.05). (3) Compared with Group I, serum triglyceride (TG) content was significantly decreased (*p* < 0.05), and serum low-density lipoprotein (LDL) content was significantly increased (*p* < 0.05) in Groups II, III, and IV. Serum total antioxidant capacity (T-AOC), superoxide dismutase (SOD), and peroxidase (PO) activities were significantly higher (*p* < 0.05) and serum malondialdehyde (MDA) content was significantly lower (*p* < 0.05) in Groups II, III, and IV compared to Group I. In conclusion, adding Schizochytrium powder to the feeding ration could affect chickens’ production performance, increase egg DHA content, and improve the antioxidant capacity of the organism. Based on the results of this study, we recommend a ratio of 1.0% Schizochytrium powder addition.

## Simple Summary

This study investigated the effects of dietary supplementation with Schizochytrium powder on production performance, egg quality, and antioxidant function in laying hens. A total of 320 hens were randomly allocated into four experimental groups: a control group fed a basal diet and three treatment groups receiving the same basal diet supplemented with 0.5%, 1.0%, or 2.0% Schizochytrium powder, respectively. The results indicated that the inclusion of Schizochytrium powder at the 2.0% level led to a reduction in both feed int conclusion, dietary supplementation with Schizochytrium powder ake and laying rate compared to the control group. However, all supplementation groups exhibited significantly enhanced eggshell strength and markedly elevated levels of docosahexaenoic acid in the eggs. Furthermore, hens receiving Schizochytrium-supplemented diets demonstrated improved antioxidant capacity, as evidenced by increased activity of key antioxidant enzymes and a notable decrease in oxidative stress markers. In conclusion, dietary incorporation of Schizochytrium powder effectively enhances the docosahexaenoic acid content of eggs and improves the antioxidant status of laying hens, although higher supplementation levels may adversely affect certain production parameters.

## 1. Introduction

As the core component of the livestock industry and an important pillar of the agri-cultural economy, increasing attention is being paid to poultry farming’s production effi-ciency and product quality, which is often improved through nutritional optimization. In addition, growing health awareness among consumers is driving the demand for func-tional foods with essential nutrients, leading to a market for products enriched with bioac-tive compounds. A potential solution lies in using natural, additive-free DHA-enriched foods to address this gap.

Schizochytrium is a unicellular spherical marine microalga that can be cultivated through heterotrophic fermentation, with characteristics including rapid cell growth, stable composition, and easy large-scale production [1]. It is rich in the omega-3 polyunsaturated fatty acid (n-3 PUFA) DHA [2,3], which promotes brain cell development and maintains retinal structure in animals [4,5,6]. Additionally, DHA helps to prevent cardiovascular and metabolic diseases, regulates gut health, and has anti-inflammatory as well as antidepressant effects in humans, benefiting people of all ages, from infants to the elderly [7,8,9]. Various governments and health organizations recommend an average dietary intake of DHA ranging from 140 to 600 mg per day [10,11]; however, DHA synthesis in the body is limited, and it cannot satisfy daily needs, so it must be supplemented through the dietary route [12], such as through deep-sea fish oil, marine microalgae, etc. [13]. It has been found that DHA-enriched eggs and other livestock products can be produced through nutritional modulation [14,15,16,17]; adding an appropriate amount of Schizochytrium powder to the diet significantly increased the DHA content of egg yolks without affecting chickens’ production performance [18,19]. In addition, Schizochytrium powder in livestock and poultry diets can promote nutrient digestion and absorption, improve pathogenic microorganism resistance, improve intestinal health, improve antioxidant capacity, and promote the healthy growth of livestock and poultry [20,21,22,23,24].

Although there have been some studies focusing on the effects of additives on poultry performance [18], there is a lack of research on the production quality and health of chickens. Therefore, in this study, we added different levels of Schizochytrium powder to chickens’ diets to investigate its effects on their performance, egg quality, and antioxidant function; to determine the appropriate addition level to chickens’ diets; and to form a nutritional formulation technology to produce DHA-rich eggs, so as to provide a scientific basis for applying Schizochytrium powder in poultry production.

## 2. Materials and Methods

### 2.1. Ethics Statement

Ethical approval for this research plan was granted by the Animal Protection and Utilization Committee of the Jiangsu Poultry Science Institute (China; approval no. JXJQS20230112).

### 2.2. Birds and Test Materials

The test chickens were from the S3 line of high-quality chickens independently bred by Jiangsu Institute of Poultry Science (a high-quality Chinese dual-purpose local breed—Shaobo chicken), which has been bred for 13 generations and has high population uniformity and excellent production and reproduction performance. The main component-related information of the Schizochytrium powder used in this study is as follows: proximate composition (dry weight basis): 25% crude protein and 40% crude fat; major fatty acids: DHA 18%, palmitic acid 35%, and docosapentaenoic acid 10%; and bioactive components: carotenoids and sterols (including ergosterol and β-sitosterol).

### 2.3. Experimental Design and Management

A total of 320 thirty-three-week-old hens with similar laying rates and body weights were selected and randomly divided into 4 groups (total of 80 in each group), with 5 replicates in each group and 16 hens in each replicate. The control group (Group I) was fed a corn–soybean meal basal diet, while the test groups were supplemented with 0.5% (Group II), 1.0% (Group III), and 2.0% (Group IV) Schizochytrium powder on top of this basal diet, respectively (Table 1). The pretest period was 1 week, and the main test period was 8 weeks. One week prior to the start of testing, the chicken house and equipment were thoroughly cleaned and disinfected; the chicken house was cleaned daily and disinfected once a week, and manure was cleared once a day, ensuring that the house was clean and tidy throughout. A negative-pressure ventilation system was adopted: through active ventilation by fans, a static pressure lower than the external environment was created inside the house, forcing fresh air to flow in uniformly and at high speed from specific air inlets, thereby achieving comprehensive air exchange and environmental control throughout the entire facility. The experimental chickens were reared in battery cages, with each one (length 60 cm × width 45 cm × height 55 cm) equipped with two nipples and one tube feeder. The indoor temperature was 23 ± 2 °C, with a relative humidity of 60 ± 5.0% and a photoperiod of 23 h, and feed and water were offered ad libitum. Mortality was recorded when it occurred. The basal diet compositions and nutrient levels are shown in Table 2.

### 2.4. Performance

Daily records were kept for each replicate group, including the number of eggs laid, total egg weight, number of non-conforming eggs (broken eggs, double-yolk eggs, sand-shell eggs, soft-shell eggs, malformed eggs, and irregularly shaped eggs), and feed intake. The following parameters were calculated for each group: egg production rate, average egg weight (AEW), non-conforming egg rate, feed conversion ratio (FCR), and ADFI. AEW = total egg weight/number of eggs. FCR = ADFI/AEW × 100.

### 2.5. Determination of Egg Quality and DHA Content in Eggs

At 42 weeks of age, hen egg quality testing began, with measurements conducted within 2 h after the hens laid eggs, and the average value over three consecutive days serving as the egg quality data for each hen, excluding malformed eggs.

Egg weight was measured using scales with an accuracy of 0.01 g, while ES was measured using an Egg Force Reader (Tenovo International Co., Ltd., Beijing, China). Then, the eggs were manually broken onto a transparent glass flat surface, and the albumen height was measured with a digital vernier caliper in the middle of the thick albumen, equidistant from the outer edge and the yolk [25]. Haugh units were subsequently calculated from the measured egg weight and albumen height as Haugh unit = 100 × log (albumen height − 1.7 × egg weight^0.37^ + 7.57) [26]. Eggshell thickness (ET) was calculated by taking the average of three points in the equatorial region and using a micrometer with a resolution of 0.001 mm. The yolk weight (YW) was determined after the chalazae were removed with forceps [27]. The eggshells were washed, air-dried, and weighed, and these measurements were used to determine the eggshell proportion (EP) (%; eggshell weight/egg weight × 100) and the yolk proportion (YP) (%; yolk weight/egg weight × 100). DHA content determination was conducted by the Nanjing Jiancheng Bioengineering Research Institute Co., Ltd. (Nanjing, China) using the following detection principle: DHA in the egg yolk is extracted from the lipid matrix using an organic solvent. The extracted lipids are then derivatized into fatty acid methyl esters (FAMEs) to increase their volatility for gas chromatographic analysis. The FAMEs are separated based on their chain length and degree of unsaturation in a GC column, and DHA is identified by comparing its retention time with an authentic standard. Quantification is achieved by measuring the peak area and comparing it to a calibration curve.

### 2.6. Determination of Serum Biochemical and Antioxidant Indices

On the last day of the experiment (42 weeks of age), 2.5 mL of blood was collected from the vein of each chicken’s wing and left to stand. The samples were centrifuged within 2 h after blood collection at 1048× *g* for 10 min to separate serum, and the supernatant was divided into 1.5 mL frozen storage tubes and stored in the refrigerator at −20 °C until use. The triglyceride (TG), low-density lipoprotein (LDL), and total cholesterol (TC) serum levels were determined using commercially available enzymatic assay kits (Nanjing Jiancheng Institute of Bioengineering, Nanjing, China) according to the manufacturers’ instructions. Briefly, for TG, the method was based on enzymatic hydrolysis of triglycerides by lipase, followed by a colorimetric reaction. For TC, the assay involved enzymatic oxidation of cholesterol-by-cholesterol oxidase, producing a colored dye. LDL cholesterol was calculated using the Fried Ewald formula (LDL = TC − HDL − TG/5). The activity of glutamic-pyruvic transaminase (GPT), also known as alanine aminotransferase (ALT), was measured using the standard UV-kinetic method, which couples the GPT-catalyzed reaction between alanine and α-ketoglutarate to the oxidation of NADH by lactate dehydrogenase, and the decrease in absorbance at 340 nm is directly proportional to the GPT activity. All absorbance readings for the colorimetric assays and kinetic measurements of GPT were performed using a microplate reader/spectrophotometer. The serum antioxidant indicators—superoxide dismutase (SOD, water-soluble tetrazolium salt-based assay), peroxidase (PO, guaiacol oxidation assay) activity, malondialdehyde (MDA, thiobarbituic acid reactive substances assay) content, and total antioxidant capacity (T-AOC, ferric-reducing ability of plasma assay)—were measured according to the method of Wang, et al. [28].

### 2.7. Statistical Analyses

After quality control processing (data that deviated more than three standard deviations from the mean were removed), the data were analyzed using one-way ANOVA with SAS 8.1 statistical software, followed by Duncan’s multiple comparison test. Orthogonal polynomial contrasts were used to evaluate the linear and quadratic effects of Schizochytrium powder supplementation levels. The data were presented as mean ± standard error of mean (SEM), and a *p*-value < 0.05 was considered statistically significant.

## 3. Results

### 3.1. Production Performance

As shown in Table 3, compared with Groups I, II, and III, Group IV showed a significant decrease in ADFI and LR (*p* < 0.05). There were no significant differences (*p* > 0.05) in other performance parameters among the four groups. The supplementation level of Schizochytrium powder showed a linear correlation with ADFI (*p* < 0.05), and both linear and quadratic correlations with LR (*p* < 0.05).

### 3.2. Egg Quality and DHA Content

As shown in Table 4, compared with Group I, ES and DHA content were significantly higher (*p* < 0.05) in Groups II, III, and IV. The DHA content was significantly higher (*p* < 0.05) in Group IV than in Groups II and III, and there were no significant differences (*p* > 0.05) in other egg quality parameters among the four groups. The addition level of Schizochytrium powder showed a linear correlation with DHA and a quadratic correlation with ES (*p* < 0.05).

### 3.3. Serum Biochemical Parameters

**Figure 1:**
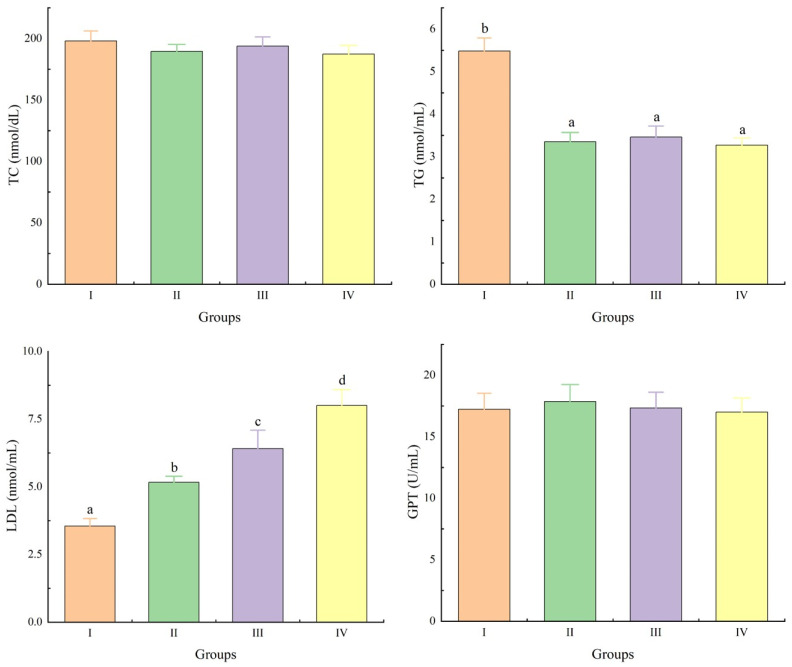
Effects of Schizochytrium powder on serum biochemical parameters in chickens. Abbreviations: TC, total cholesterol; TG, triglyceride; LDL, low-density lipoprotein; GPT, glutamic-pyruvic transaminase. Data marked with a different letter differ significantly (*p* < 0.05).

As shown in Figure 1, compared with Group I, the serum TG content in Groups II, III, and IV was significantly decreased, while the LDL content was significantly increased (*p* < 0.05). Among them, the LDL levels in Groups II, III, and IV showed a significant increasing trend (*p* < 0.05). No significant impact on TC and GPT (*p* > 0.05).

### 3.4. Antioxidant Indices

**Figure 2:**
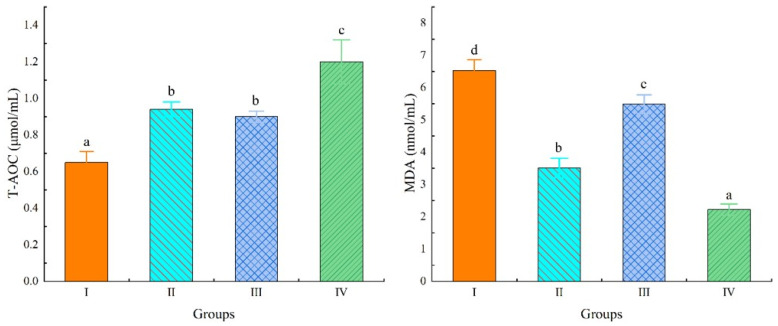
Effects of Schizochytrium powder on serum T-AOC and MDA content in chickens. Abbreviations: T-AOC, total antioxidant capacity; MDA, malondialdehyde. Data marked with a different letter differ significantly (*p* < 0.05).

**Figure 3:**
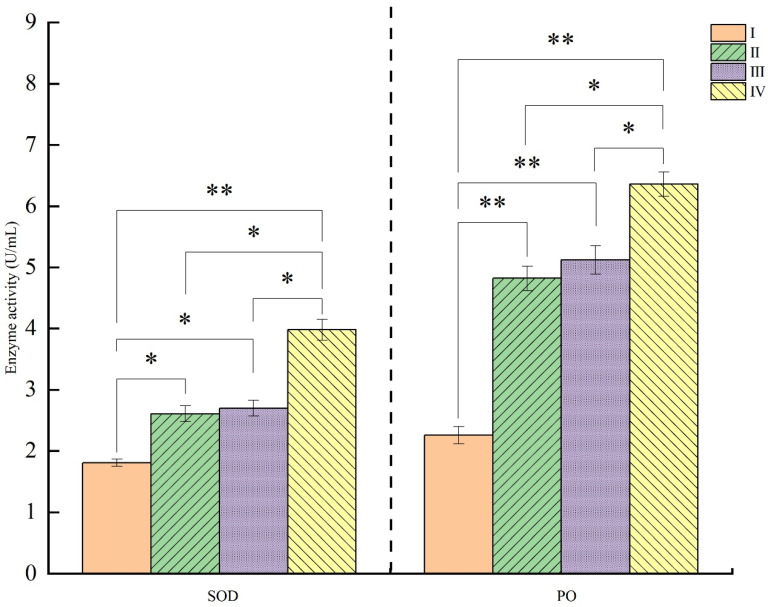
Effects of Schizochytrium powder on serum SOD and PO activities in chickens. Abbreviations: SOD, superoxide dismutase; PO, peroxidase. Significant differences are marked as * 0.01 < *p* ≤ 0.05, ** *p* < 0.01.

As shown in Figure 2 and Figure 3, compared to Group I, the T-AOC content and SOD and PO activities were significantly increased in Groups II, III, and IV, with Group IV being significantly higher than Groups II and III (*p* < 0.05). The MDA content in Group III was significantly lower than that in Group I, and significantly higher than that in Groups II and IV (*p* < 0.05).

## 4. Discussion

The inclusion of novel feed ingredients, particularly those derived from sustainable marine sources, represents a significant area of modern poultry nutrition research aimed at enhancing productivity and sustainability. The most compelling finding of this study is that, compared to Groups I, II, and III, Group IV exhibited a significant decrease in ADFI and LR, while no significant changes were observed in AEW and FCR, a result similar to some of Curabay, et al. [29]’s findings, who indicated that adding Schizochytrium to the diet of chickens had no significant effect on AEW or FCR. But, according to research by Wang et al. [19] and Kiran et al. [18], adding Schizochytrium powder to the diet had no effect on the ADFI of Hy-Line Brown chickens. The discrepancy in these results may be attributed to differences in the breed or age of the hens studied. A decrease in ADFI can be attributed to several factors: Firstly, the high lipid content of Schizochytrium powder alters the dietary energy density. Although the dosage used in the experiment was very low, the resulting increase in metabolizable energy may have led to a natural reduction in voluntary feed intake when birds attempted to regulate their energy expenditure—a well-documented phenomenon in poultry. Secondly, and more probably, palatability may be a factor; marine-derived ingredients can impart specific flavors or odors to the feed that may be less appealing to chickens, leading to neophobia (a reluctance to eat novel foods) or simply a reduced preference. This effect often becomes pronounced only after surpassing a certain inclusion level, which aligns with the fact that this significant effect was only seen in Group IV, which presumably received the highest dose. The concomitant decrease in LR in Group IV is almost certainly a direct consequence of the reduced energy and nutrient intake resulting from lower ADFI. Chickens are precision-fed to meet their exact nutritional requirements for maintenance and production. Any substantial drop in feed consumption disrupts this balance, forcing the birds to prioritize nutrient partitioning. Energy is first diverted to vital maintenance functions, often at the expense of reproductive performance. Therefore, the observed decline in egg production is not necessarily a direct pharmacological effect of the Schizochytrium powder itself, but rather a secondary effect mediated through reduced nutrient availability. This underscores the importance of dose-dependent studies, as it suggests there may be an optimal inclusion level (represented by Group III) that provides benefits without suppressing intake and production.

It was found that adding 1.0% Schizochytrium powder to the diet significantly increased n-3 polyunsaturated fatty acid accumulation in egg yolks, especially DHA [30], which could be converted into high-value-added nutritious egg products. Furthermore, a study by Wang et al. [19] showed that adding 2% Schizochytrium powder and 1000 mg/kg choline to feed significantly increased the DHA levels in chicken egg yolks. Egg quality is one of the most important indicators of chickens’ performance, which directly reflects the nutritional status, health status, and feeding management level of chickens. Through measuring egg quality, the production status of chickens can be understood in a timely manner, potential problems can be identified, and appropriate measures can be taken to solve issues. In this experiment, the effects of Schizochytrium powder on the quality of chickens’ eggs were mainly manifested in ES and DHA content. ES and DHA content were significantly higher in all experimental groups than in the control group, and DHA content significantly increased sequentially, which was consistent with the above study, in which the enhancement of ES provided better performance for the subsequent incubation of chickens. This may be due to the positive effects of DHA on chicken metabolism, which promotes the absorption and utilization of nutrients. However, in the present study, we focused on chicken egg quality after adding Schizochytrium powder but did not carry out an in-depth study on chickens’ breeding performance and metabolic regulation after adding Schizochytrium powder. Therefore, in the future, systematic measurements of the fertilization rate and hatchability of hatching eggs from chickens will be conducted to comprehensively evaluate the effects of Schizochytrium powder on reproductive performance, hatching performance, and physiological health. This will provide more scientific evidence and technical support for Schizochytrium powder use in the breeding strategies and production efficiency of chicken farmers.

The significant decrease in serum triglyceride (TG) content across all treatment groups (II, III, IV) compared to the control (Group I) suggests that *Schizochytrium* powder actively influences lipid metabolism. This is consistent with the findings of Park, et al. [31]. This hypolipidemic effect is likely attributable to its rich composition of polyunsaturated fatty acids (PUFAs), particularly docosahexaenoic acid (DHA). PUFAs are known to enhance fatty acid β-oxidation and suppress lipogenic enzyme activity, thereby reducing circulating triglyceride levels [9]. Conversely, the significant increase in serum low-density lipoprotein (LDL) content was an unexpected outcome; typically, dietary PUFAs are associated with a favorable lipid profile, including reduced LDL [10,32]. This result may be related to the specific cholesterol transport mechanisms in birds, which differ from those in mammals, or to the complex interplay between different lipid fractions. The absence of a significant effect on TC further underscores the complexity of this modulation and indicates that the changes are specific to certain lipoprotein classes rather than overall cholesterol homeostasis. The lack of impact on GPT activity is a crucial finding, as it indicates that the lipid parameter alterations were not associated with hepatic damage, affirming the safety of the supplementation at the administered levels.

The significant increase in total antioxidant capacity (T-AOC), superoxide dismutase (SOD), and peroxidase (POD) activities in Groups II, III, and IV directly confirms the hypothesis that *Schizochytrium* powder improves antioxidant status [32]. SOD and POD are first-line enzymatic antioxidants that catalyze the neutralization of superoxide anions and hydrogen peroxide, respectively. Their elevated activity signifies a strengthened capacity to mitigate oxidative damage. The fact that Group IV exhibited superior activity compared to Groups II and III suggests that the antioxidant response is directly correlated with the powder inclusion rate. This enhancement may be related to the high DHA content or other specific phenolic compounds; while PUFAs are susceptible to peroxidation, they also act as signaling molecules that can upregulate the expression of antioxidant enzymes via transcription factors [33]. This interpretation is further supported by the data on malondialdehyde (MDA), a key marker of lipid peroxidation [30]. The significant reduction in MDA content in Group III compared to the control reflects a decrease in oxidative damage to cellular lipids; however, the finding that its level in Group III was higher than in Groups II and IV presents a complex, non-linear relationship that may indicate an optimal dosage range (perhaps in Group IV) for maximizing antioxidant protection and minimizing peroxidation. In conclusion, dietary Schizochytrium powder effectively enhances the antioxidant capacity of chickens and modulates their lipid metabolism, establishing its promise as a valuable dietary intervention for improving animal health and productivity.

In summary, the findings reveal that Schizochytrium powder can improve certain economic indicators without compromising key production performance, while also highlighting its potential as a functional feed ingredient for modulating oxidative stress and lipid metabolism. It should be noted that microalgae supplementation was performed on top of the control diet, which may have caused minor dilution of other dietary components. While this design was necessary to isolate the effects of microalgae, the potential influence of this nutrient shift should be considered when interpreting the results.

## 5. Conclusions

In conclusion, dietary supplementation with Schizochytrium powder can increase egg DHA content, while the improvement in ES enhances the eggs’ resistance to pressure. Additionally, it boosts the antioxidant capacity of chickens, thereby strengthening their overall resistance. However, excessive supplementation may reduce certain egg production performance metrics; for instance, when the supplementation level reaches 2.0%, the LR decreases significantly. Under the conditions of this experiment, it is recommended to add Schizochytrium powder to the diet at a 1.0% level.

## Figures and Tables

**Table 1:** Schizochytrium powder supplementation in basal diet.

Groups	Treatment Plan
Group I	Control group with basal diet
Group II	Basal diet with 0.5% Schizochytrium powder
Group III	Basal diet with 1.0% Schizochytrium powder
Group IV	Basal diet with 2.0% Schizochytrium powder

**Table 2:** Composition and nutrient levels of basal diet (air-dry basis).

Ingredients	Content	Nutrient Levels ^2^	Content
Corn, %	64.75	Metabolic energy, (MJ/kg)	11.09
Soybean meal, %	24.30	Dry matter, %	87.60
*DL*-methionine, %	0.08	Crude protein, %	15.50
*L*-lysine·HCl, %	0.07	Calcium, %	3.50
Limestone, %	9.10	Available phosphorus, %	0.30
CaHPO_4_, %	1.00	Methionine, %	0.33
NaCl, %	0.38	Sulfur amino acids, %	0.59
Premix ^1^, %	0.22	Lysine, %	0.80
Choline chloride, %	0.10		
Total, %	100.00		

^1^ The premix provided the following per kg of the diet: VA, 8000 IU; VD_3_, 1000 IU; VE, 20 IU; VK_3_, 0.50 mg; VB_1_, 2.00 mg; VB_2_, 8.00 mg; VB_6_, 3.50 mg; VB_12_, 10.00 µg; nicotinic acid, 35.00 mg; *D*-pantothenic acid, 10.00 mg; folic acid, 0.55 mg; biotin, 0.18 mg; Cu (as copper sulfate), 10 mg; Fe (as ferrous sulfate), 80 mg; Mn (as manganese sulfate), 80 mg; Zn (as zinc sulfate), 75 mg; I (as potassium iodide), 0.40 mg; Se (as sodium selenite), 0.30 mg. ^2^ Metabolic energy, available phosphorus, and amino acids were calculated values, while the others were measured values.

**Table 3:** The effects of Schizochytrium powder on the performance of chickens.

Items	Groups				*SEM*	*p*-Value		
	I	II	III	IV		ANOVA	Linear	Quadratic
ADFI, g	119.93 ^b^	118.11 ^b^	117.73 ^b^	109.52 ^a^	0.284	0.035	0.018	0.064
AEW, g	42.61	41.97	42.43	41.17	0.061	0.105	0.452	0.677
FCR	2.81	2.81	2.77	2.66	0.015	0.073	0.325	0.273
LR, %	80.56 ^b^	80.35 ^b^	79.05 ^b^	77.91 ^a^	0.649	0.031	0.020	0.014

Means in the same row followed by different letters are significant (at *p* = 0.05). Abbreviations: ADFI, average daily feed intake; AEW, average egg weight; FCR, feed to egg ratio; LR, laying rate.

**Table 4:** The effects of Schizochytrium powder on egg quality of chickens.

Items	Groups				*SEM*	*p*-Value		
	I	II	III	IV		ANOVA	Linear	Quadratic
ES, kg/cm^2^	40.13 ^a^	42.76 ^b^	42.44 ^b^	42.66 ^b^	0.076	0.032	0.101	0.021
ET, mm	0.32	0.31	0.32	0.32	0.001	0.256	0.321	0.422
EP, %	10.31	10.71	10.67	10.77	0.023	0.682	0.261	0.312
YP, %	32.42	32.61	32.40	32.43	0.079	0.754	0.566	0.453
AH, mm	5.76	5.80	5.86	5.87	0.009	0.562	0.498	0.397
HU	82.60	82.50	82.67	82.57	0.077	0.236	0.413	0.564
DHA, mg/kg	0.90 ^a^	2.31 ^b^	2.58 ^b^	3.61 ^c^	0.005	0.001	0.001	0.122

Means in the same row followed by different letters are significant (at *p* = 0.05). Abbreviations: ES, eggshell strength; ET, eggshell thickness; EP, eggshell proportion; YP, yolk proportion, AH, albumen height; HU, haugh units; DHA, docosahexaenoic acid.

## Data Availability

The data in this study can be obtained upon reasonable request to the corresponding author.
